# Potential uses of AI for perioperative nursing handoffs: a qualitative study

**DOI:** 10.1093/jamiaopen/ooad015

**Published:** 2023-03-16

**Authors:** Christopher Ryan King, Ayanna Shambe, Joanna Abraham

**Affiliations:** Department of Anesthesiology, Washington University School of Medicine, Washington University in St. Louis, St. Louis, Missouri, USA; Department of Anesthesiology, Washington University School of Medicine, Washington University in St. Louis, St. Louis, Missouri, USA; Saint Louis University School of Medicine, St. Louis, Missouri, USA; Department of Anesthesiology, Washington University School of Medicine, Washington University in St. Louis, St. Louis, Missouri, USA; Institute for Informatics, Washington University in St. Louis, St. Louis, Missouri, USA

**Keywords:** artificial intelligence, postoperative nursing, PACU, handoffs, situational awareness

## Abstract

**Objective:**

Situational awareness and anticipatory guidance for nurses receiving a patient after surgery are keys to patient safety. Little work has defined the role of artificial intelligence (AI) to support these functions during nursing handoff communication or patient assessment. We used interviews to better understand how AI could work in this context.

**Materials and Methods:**

Eleven nurses participated in semistructured interviews. Mixed inductive-deductive thematic analysis was used to extract major themes and subthemes around roles for AI supporting postoperative nursing.

**Results:**

Five themes were generated from the interviews: (1) nurse understanding of patient condition guides care decisions, (2) handoffs are important to nurse situational awareness, but multiple barriers reduce their effectiveness, (3) AI may address barriers to handoff effectiveness, (4) AI may augment nurse care decision making and team communication outside of handoff, and (5) user experience in the electronic health record and information overload are likely barriers to using AI. Important subthemes included that AI-identified problems would be discussed at handoff and team communications, that AI-estimated elevated risks would trigger patient re-evaluation, and that AI-identified important data may be a valuable addition to nursing assessment.

**Discussion and Conclusion:**

Most research on postoperative handoff communication relies on structured checklists. Our results suggest that properly designed AI tools might facilitate postoperative handoff communication for nurses by identifying specific elevated risks faced by a patient, triggering discussion on those topics. Limitations include a single center, many participants lacking of applied experience with AI, and limited participation rate.

## BACKGROUND AND SIGNIFICANCE

Inpatient handoffs are the transfer of responsibility, information, and control between clinicians or teams. Incomplete or inaccurate handoffs are a source of subsequent medical errors and patient injury,^1–3^ particularly for patients undergoing major surgery.^4–7^ We focus on postoperative nurse handoffs during surgical patient transfers from the operating room (OR) to the postanesthesia care unit (PACU) and from the PACU to inpatient ward. Handoffs are important for receiving nurses to understand the patient's situation because residual sedation, pain, delirium, fatigue, and surgical injuries can make patient-nurse communication difficult. Additionally, the patient's context changes; surgery eliminates some concerns and creates the opportunity for new complications. The data surrounding surgical patients are voluminous and diverse while simultaneously incomplete, which strains the ability of receiving nurses to review and assimilate it *de novo*.^8–10^ Two functions of handoff are of special interest to us: situational awareness and anticipatory guidance. Situational awareness is the combination of perceiving critical factors in the environment, understanding what those factors mean for the clinician's goals, and understanding what will happen next.^11^ Anticipatory guidance is the communication of likely patient status changes and plans for how to address them.^12^^,^^13^ These 2 functions support early recognition and coordinated treatment of complications, which have substantial effects reducing postoperative mortality and morbidity. ^14^ Major handoff quality improvement projects have integrated both of these concepts.^15–17^ Protocols and checklists are employed to ensure that key information is transmitted during handoffs throughout healthcare.^18–20^ Some electronic health records (EHRs) have integrated standardized handoffs,^21^ including nurse-to-nurse handoffs^15^^,^^22^ and perioperative nursing handoffs specifically.^23^^,^^24^ Nevertheless, handoff-related information gaps are common for postoperative patients.^10^^,^^25–28^

The EHR has promise for mitigating and reducing these information gaps. EHRs place an enormous amount of data at the fingertips of all clinicians. In theory, this ought to allow a nurse to prepare for handoff and recover from an incomplete handoff. Dashboard-type displays can be used during handoffs for this summary function. ^29^ Despite this promise, most handoff-EHR integration work does not focus on the critical functions of situational awareness and anticipatory guidance.^30^ Staggers et al^31^ found that existing EHR handoff summaries were too rigid and incomplete to be useful; additionally, they interfered with the receiving nurse's encoding of information via note taking. They subsequently found that nurses made little use of EHR handoff support due to these limitations.^32^ Calculations and displays of EHR data can be viewed as sense-making, with tension between different purposes and users.^33^

Artificial intelligence (AI) integrated into EHRs is an exciting, related development. AI is a broad term, including all computer programing which replicates or imitates cognitive functions. The most common approach applying AI to EHR data for nursing is supervised machine learning (ML), in which algorithms use EHR data as inputs to predict unknown or unrecorded characteristics of a patient, such as future adverse events, current patient condition, or undocumented comorbidities.^34^ Although often discussed exchangeably, ML (an approach to pattern recognition) and clinical decision support (CDS) (applying pattern recognition to suggest actions or documentation) are conceptually different. For a given AI/ML pattern recognition tool, a wide variety of uses cases, visualizations, and user interfaces are possible. AI using EHR data has become much more general and accurate in the last few years,^35^^,^^36^ allowing prediction of perioperative events^37–41^ and learning effective treatment strategies.^42^ AI is able to interpret nursing documentation to recognize patient types and predict clinical deterioration.^43–47^ Research has explored AI/ML in several roles to augment the capabilities of bedside nurses, including identifying care needs or predicting adverse events based on EHR data, scheduling and equipment management, patient activity tracking, processing nursing documentation for transitions of care, quantifying risks in family discussions, and interactive patient education.^34^^,^^48–50^ For example, ML identification of patients with a high risk of pressure ulcers^51^^,^^52^ or falls^53^ can trigger CDS for nursing interventions. The related CDS literature for nurses has focused on recommending specific actions based on scoring systems and expert-devised rules.^54^ In addition to predicting adverse events, AI/ML models can flag important data for review. While information dashboards have long been integrated into EHRs with expert-driven rules for abnormal data,^31^^,^^32^^,^^55–57^ contemporary systems include AI/ML models to identify “relevant” patient data.^58–60^

Very few AI studies have gone beyond initial development phases or shown benefits to stakeholders,^49^^,^^50^ and the more developed use-cases are often highly specialized, such as rapid-response-team alarms. ^48^ Expanding nursing engagement in design of AI projects is a recognized priority,^61^ as very few AI or information system studies involve nurses at early stages.^50^^,^^62^

A handful of studies have considered the impact of AI prediction in augmenting handoff communication. In the neonatal ICU context, Hunter et al^63^ used natural-language generation to summarize EHR data and generate potential problems and care plans in a dynamic shift-change report. Forbes and colleagues^56^^,^^64^ envisioned a dynamic EHR integrated shift-report summary for nurses including key data, diagnoses, and predicted adverse events. Hunter and Forbes's work^56^^,^^63^^,^^64^ suggests a distinct role for AI prediction from traditional CDS: facilitating problem-based report and assessment during handoffs. Although clinician assessment of the patient's condition is a key part of all structured handoffs, AI identification of likely complications and important data integrated into dynamic “handoff sheets” could supplement handoff assessment more flexibly than traditional checklist-based protocols.

We previously explored related ideas at the OR to intensive care unit handoff, which often has a brief nurse-to-nurse component due to the multidisciplinary nature of the handoff.^65^^,^^66^ Key findings of that study were the difficulty of making EHR information universally accessible, the need to focus on AI with direct relevance to patient care, and general acceptance of blending AI risk prediction with current summaries of patient data into a handoff tool. However, the ICU shift-change and OR-ICU handoffs previously studied are quite different from the OR-PACU-ward transition.

## OBJECTIVES

Although direct experimentation with implementing AI support for perioperative handoffs would be informative, we set out to establish a use-case with clinicians and refine what content would be useful for clinicians prior to implementation. We identified 3 unanswered preliminary questions in prior research about postoperative bedside nurses as givers or receivers of handoff which we aim to address: (1) would postoperative nurses accept AI recommendations for handoff topics? (2) would nurses find AI-based predictions of adverse events useful and relevant? (3) would a single presentation of AI-based predictions be acceptable to most nurses? The goal of this single-center qualitative study was to explore these topics and how AI added to a handoff workflow might fit into the situational awareness, assessment, monitoring, and communication goals of postanesthesia care unit (PACU) and postoperative ward nurses. We intend these findings to guide subsequent design and implementation efforts, but we did not evaluate a specific AI product or technical implementation.

## MATERIALS AND METHODS

Our research included 2 activities: direct observation of handoffs to establish context in the research team and interviews with postoperative nurses to directly address the research questions.

### Setting

Barnes-Jewish Hospital is a 1400-bed academic medical center in St Louis, Missouri. We focused on the Acute and Critical Care Surgery (ACCS) division, which performs approximately 1600 inpatient surgeries annually, primarily trauma, and acute abdominal surgery. All postoperative patients (other than those directly admitted to intensive care) recover from anesthesia in the PACU, a 30-bed area. Four hospital units subsequently care for ACCS patients: 2 dedicated hospital wards and 2 high-dependency units. The high-dependency units are shared with otolaryngology, abdominal organ transplant, and hepatobiliary services.

### Observations

Researchers selected surgical cases for direct observation from the OR schedule based on the primary surgery service (ACCS). We also included patients likely to be admitted to high-dependency units based on their procedures. We attempted observation on all cases meeting these criteria between 9 am and 5 pm on weekdays. Researchers conducted direct observations under Washington University IRB approval (#201812137 and #202009066) with the consent of the PACU nurse to shadow their interactions with other clinicians (OR circulator nurse, anesthesia clinician, surgery clinician, and wards nurse) and recorded notes following a structured outline.^67^ The IRB approved verbal consents with electronic provision of study information as a replacement for written consents during the coronavirus disease 2019 pandemic. Because we performed these observations to provide interpretative context for interview analysis rather than directly answer study questions, we do not separately report findings from observations. We include this description only to report the nurse participant recruitment process.

### Description of perioperative handoff processes and care teams

**Figure 1.**
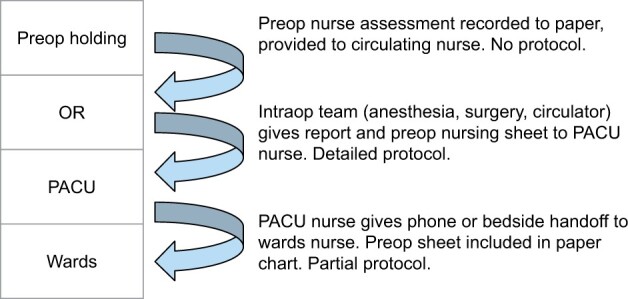
Illustration of perioperative handoff stages.

Figure 1 illustrates the handoff process. Prior to surgery, a preoperative holding area nurse completes a health status inventory in the Epic EHR and on a paper record (Supplementary Appendix S1) which is passed to PACU. The preoperative nurse and OR circulating nurse complete an informal handoff. After surgery, a surgery resident or fellow, the OR circulating nurse, and an anesthesia clinician transport the patient to PACU. OR to PACU handoff follows a protocol (Supplementary Appendix S1), where the circulating nurse, surgeon, and anesthetist each give handoff to the PACU nurse. The handoff sheet (Supplementary Appendix S1), consent documents, and backup records from surgical implants, and blood transfusions are the only common paper records. All other documentation is electronic.

Once PACU staff and the supervising anesthesiologist deem a patient ready to leave the PACU, the PACU nurse gives handoff to the ward nurse either at the bedside (high dependency unit) or by phone call (ward units). A guideline addresses the handoff between PACU and the wards nurses (Supplementary Appendix S1). Fellows, resident physicians, nurse practitioners, and the attending surgeon jointly manage postoperative patients. The nurse practitioner or resident physician implementing ward care is not directly involved in the surgery. We refer to that resident or nurse practitioner as the *midlevel clinician*.

### Interview participants and data collection

Concurrently with our direct observations, we recruited a convenience sample of nurses from the PACU, ACCS wards, and high-dependency units. We chose interviews instead of focus groups to allow us to hear multiple independent perspectives, and for pragmatic reasons. During the study period, nurse participants faced high workloads, making scheduling focus groups difficult. We conducted interviews under Washington University IRB approval (#201812137 and #202009066) with the consent of the participant. Authors King and Shambe conducted interviews using the same guide (Supplementary Appendix S2). The content of the interviews focused on handoff communication, patient assessment, physician communication, and potential roles for AI. We conducted interviews over the phone or voice application with audio recording, which was transcribed verbatim.

### Analysis

Two researchers (King and Shambe) double-coded interviews using a mixed inductive-deductive reflexive thematic analysis approach. First, we familiarized ourselves with the data by reviewing the transcripts and fragmenting them into topical sections. Second,^68^ we organically generated open codes after the first review. We applied deductive codes based on relevance to major study questions (listed in Supplementary Appendix S3). We labeled each statement as relevant to OR-PACU or PACU-ward handoffs based on the surrounding context. Next, the coders discussed the set of open codes and resolved conflicts by consensus. We generated initial subthemes from groups of related codes. We then compared OR-PACU and PACU-ward coded data for similar subthemes that could be coalesced. We did not formalize a codebook, but we returned to the raw statements for consistency with the subthemes and examined them for relationships to other identified subthemes. We then jointly refined subthemes based on recoded data and clustered subthemes into themes based on connecting stories. At each stage, coders compared codes and resolved disagreements. The coders and a third researcher (Abraham) reviewed and revised themes. After the construction of the coding tree, coders checked statements to validate their applicability to the higher-level themes. After 10 interviews, we completed a first round of coding, and we found that most topics were addressed by multiple participants, meaning that saturation was likely; we found no new topics during analysis of the 11th interview and stopped recruitment.

Supplementary Appendix S4 is a consolidated criterion for reporting qualitative research (COREQ) checklist,^69^ a qualitative research reporting framework, along with some additional methods details.

## RESULTS

We conducted 11 total interviews: 7 PACU nurses and 4 ward nurses. Supplementary Table S5 (Supplementary Appendix S5) displays the 5 major themes in our findings, subthemes, and exemplar quotations of each subtheme: (1) nurse understanding of patient condition guides care decision; (2) handoffs are important to nurse situational awareness, but multiple barriers reduce their effectiveness; (3) AI may address barriers to handoff effectiveness; (4) AI may augment nurse care decision making and team communication outside of handoff; and (5) EHR user experience and information overload are likely barriers to using AI during handoffs. These themes had substantial interactions, and with each subtheme, we note closely related subthemes. Supplementary Table S5 shows the relevance to OR-PACU, PACU-ward, or both handoffs of each sub-theme along with number of interviews referencing each.

### Nurse understanding of patient condition guides care decisions

Participants stressed that their bedside presence allowed rapid detection and hopefully mitigation of complications. They universally agreed that their understanding of the issues facing a patient modified what signs and symptoms they were alert for (Subtheme 1.b), what issues they communicated to the PACU or midlevel clinician (Subtheme 1.c), and what treatments they recommended. Several participants stated that although almost all treatment changes required a team discussion, their recommendations were likely to be considered or acted on.

### Handoffs are important to nurse situational awareness, but multiple barriers reduce their effectiveness

Participants stressed that accurate handoff was a critical way to learn about the patient's state, expectations for recovery, and needs in the high-turnover environment of PACU (Subtheme 2.a). However, they acknowledged barriers where the documentation they relied on was incomplete (Subtheme 2.d), the handoff-giver did not know the relevant information, or they did not understand what needed to be conveyed. Participants agreed that problem-focused handoffs with anticipatory guidance were extremely useful, but that many topics in handoffs were not relevant or recited data without context (Subtheme 2.b). Closely related to this concern was a lack of shared priorities between the handoff giver and receiver. It was frequent for participants to describe receiving handoffs focusing on details they found to be irrelevant or unintelligible, and for handoff, participants to not value topics on which their counterparty asked questions (Subtheme 2.c).

### AI may address barriers to handoff effectiveness

Several participants commented on how AI risk prediction at handoff might mitigate mismatch between handoff givers and receivers. First, almost all participants agreed that if AI identified a patient at high risk for a complication, that this topic would be prioritized for discussion at handoff, and that those receiving handoff would ask follow-up questions regarding the patient state and the current plan (Subtheme 3.a). Second, a high calculated risk could alert them that a known comorbidity was more severe than they expected (Subtheme 3.b), which was information frequently absent from documentation. Third, awareness that a patient was overall high-risk would prompt nurses to closely review all available data and prioritize shared careful patient evaluation (Subtheme 3.c). Finally, automatic identification of EHR data elements which increased the patients’ risk could mitigate data omissions, especially if that data was in an unusual location (Subtheme 3.d). Although several participants gave examples of how they might relate data given at handoff to specific AI-identified problems (ameliorating the laundry-list type handoff of Subtheme 2.c), none explicitly identified using the AI-identified problems to organize data.

### AI may augment nurse care decision-making and team communication outside of handoff

PACU handoff is a critical time for establishing joint plans and midlevel clinician communication needs; however, posthandoff communication was also regarded as important. Ward participants noted that midlevel clinicians rarely proactively contacted them, leaving nurses to deduce what issues required communication or nursing action (Subtheme 4.a). Some participants noted that AI could help target posthandoff nurse-midlevel communication in 2 ways. First, if a patient had been identified as high risk, the resistance to contacting the midlevel clinicians to discuss that topic would be lowered (Subtheme 4.b). Second, the nurse's holistic view of patient risk might be difficult to communicate, and AI-based pattern matching would make this more concrete and easier to request midlevel clinicians act on or personally evaluate.

Participants noted incomplete midlevel clinician documentation and other EHR information negatively affected their independent assessment of the patient (Subtheme 4.c). AI identification of *alternative* key data would then be valuable. Additionally, AI-identified risks for adverse events would allow the nurse to better target their assessment and monitoring independent of any effect on handoff (Subtheme 4.d). Participants noted that AI-identified elevated risks could allow them to target interventions within their scope of practice, such as fall prevention, delirium prevention, and pneumonia prevention (Subtheme 4.d). Multiple participants endorsed the desire for more accurate prediction of patients likely to require higher nursing workload or ICU transfer, which they could use to allocate their resources.

### EHR user experience and information overload are likely barriers to using AI

Participants identified several barriers for nursing use of AI, largely centered around the user experience and the potential for excessive information volume. First, because of the large number of different methods for accomplishing most tasks in Epic, participants did not recommend the same locations for viewing AI risk prediction. Second, preferred visualizations also differed between participants, with participants variously endorsing absolute risk estimates, relative risks, simplified high-medium-low risk flags, and plots. Several participants noted that existing clinical decision support and alerts already generate alarm fatigue, and that additional flags would likely be ignored unless they had high value (Subtheme 5.c). Finally, participants noted the potential for information overload with more complex outputs (Subtheme 5.d).

## DISCUSSION

Our interviews highlighted the importance of team communication and anticipatory guidance at and around postoperative handoffs for nurses to optimize patient care. The data gave consistent answers to our knowledge-gap questions:

1. *Would postoperative nurses accept AI recommendations for handoff topics?* Yes, participants believed that AI which identified patients at elevated risk would lead to focused handoff communication and physician-nurse team communication on those topics, increasing anticipatory guidance and situational awareness. Nurses overall expressed little hesitance to include AI-estimated risks in their handoff assessments.
2. *Would nurses find AI-based predictions of adverse events useful and relevant?* Yes, participants believed that well-functioning AI risk assessment would lead to activating nurse-driven interventions, allocating resources (such as high-dependency beds) more efficiently, and prioritizing monitoring for higher-risk outcomes. To accomplish this, participants desired both overall measures of acuity and estimation of a broad collection of risks.
3. *Would a single presentation of AI-based predictions be acceptable to most nurses?* No, participants acknowledged diverse methods of using the EHR, and diverse preferences for information presentation. While our participants were enthusiastic for AI identification of relevant information in the EHR, they also acknowledged barriers surrounding the user experience of adding AI to their workflows and the potential for information overload. The ability to easily integrate AI into multiple EHR workflows and choose a personalized presentation will be necessary for it to succeed.

Our work contrasts with much of the development of EHR AI support for nurses,^54^ which largely focuses on medication documentation, medication administration, and very simple rule-based systems to identify specific nursing needs. Our work also highlights the need for handoff communication to adapt to the patient's condition, contrasting with the dominant theme of the literature for improving handoffs: standardized communication and checklists.^70^ Several small studies from other nursing contexts have found similar themes. Home care nurses in a prior study expressed a similar use case for AI to modify the intensity of their services but did not discuss its role in transitions of care.^71^ User-design work for EHR-integrated shift-change handoff support had similar ideas, arriving at a design which blended data and predictive risks.^56^^,^^64^ Although their work stemmed from interactions with nurses and nursing students, their manuscripts do not give enough methods details to further explore similarities with our work. Nurse users largely accepted a prototype system for shift change in the neonatal intensive care unit which focused on summarizing data in natural language and included expert decision rules as a minor component.^63^

Our findings can also be related to work with dashboards intended to detect change in patient status which lack explicit AI predictions.^54^ In our work on OR to ICU handoffs,^66^ participants endorsed similar desires to integrate AI into summaries of patient data like laboratory results and vital signs and the need to focus on actionability. In contrast to ICU participants, our participants felt that AI augmentation of handoff topics could be useful, AI assessment of risks for midlevel clinician communication would be valuable, and that AI could assist their selection of necessary patient assessment steps. Very recently, experience with risk-predicting AI suggests that it facilitates a shared mental model and coordination across disciplines by providing a reference point for patient status,^72^ including using this shared reference point for escalation of care.^73^ Our participants echoed this idea in Subtheme 4.b.

Similar to others,^73–75^ we found that extraction of directly interpretable patient data and actionable needs was a high priority (Subtheme 4.d). Prior work has also found that nurses more frequently use a “bottom-up” (data and needs first) approach to patient summarization,^76^ which agrees with our finding of specific risk-increasing data and conditions being important for handoff support (Subtheme 3.c). Physicians and nurses rate explainability in terms of patient data and personal understanding as highly related to trust in AI;^73^^,^^77^ however, current methods of AI explainability have been found to have limited usefulness in practice.^78^ Some implementation studies have found that AI-based alerts are relatively more salient to nurses than physicians in this regard.^79^ Imperative AI-based CDS has been effective in some direct use cases, supporting this approach,^80^ but it runs the risk of automation bias.^81^^,^^82^ Similar to the findings of others,^73^ our participants indicated that they would consider the AI as a suggestion of where to start an evaluation rather than a prescriptive mandate (Subtheme 4.d).

Taken together, our findings and these prior studies suggest that AI can support nurses in their more general cognitive tasks, and that future AI design efforts should (1) target critical moments of evaluation like shift change and handoff and (2) incorporate estimates of acuity, condition severity, and influential data outside narrow “nursing related” problems. We anticipate that an adaptive handoff sheet design like Hunter and Forbes’s work^56^^,^^63^^,^^64^ containing automated identification of problems relevant to each patient and data pertinent to those problems will emerge from further research with this population and ongoing technical testing. This optimism is restrained by the many practical implementation difficulties that plague clinical AI,^83^ which was echoed in the concerns of our participants (Subtheme 5).

### Limitations

Our study drew participants from a single center, which limits the range of experiences and exposure to alternative EHRs. The ward nurses worked in a small number of units, limiting the generalizability. The number of participants and recruitment rate from those potentially eligible were both low. The participants had limited experience with AI, which limits the reliability of the findings. The setting was an academic medical center, so the views may not reflect the experiences of those outside this type of setting. Our interview was semistructured, and participants were informed on the nature of our study. They may have endorsed ideas to be agreeable, but participants seemed to feel free to disagree.

## CONCLUSION

This interview study of perioperative nurses at an academic medical center found that participants were receptive to AI as a potential adjunct for postoperative handoff communication. Ongoing studies will evaluate the usability and communication impact of AI tools in nursing practice.

## Supplementary Material

ooad015_Supplementary_Data

## Data Availability

Data cannot be shared for ethical/privacy reasons. The data underlying this article cannot be shared publicly due to identified discussions of clinician behavior and patient stories. Participants were offered privacy to allow them to openly share concerns about their workplace. The data will be shared on reasonable request to the corresponding author and IRB approval.

## References

[1] Keebler JR , Lazzara EH, Patzer BS, et al Meta-analyses of the effects of standardized handoff protocols on patient, provider, and organizational outcomes. Hum Factors 2016; 58 (8): 1187–205. 27821676 10.1177/0018720816672309

[2] Solet DJ , Norvell JM, Rutan GH, Frankel RM. Lost in translation: challenges and opportunities in physician-to-physician communication during patient handoffs. Acad Med 2005; 80 (12): 1094–9. 16306279 10.1097/00001888-200512000-00005

[3] Humphrey KE , Sundberg M, Milliren CE, Graham DA, Landrigan CP. Frequency and nature of communication and handoff failures in medical malpractice claims. J Patient Saf 2022; 18 (2): 130–7. 35188927 10.1097/PTS.0000000000000937

[4] Segall N , Bonifacio AS, Schroeder RA, et al; on behalf of the Durham VA Patient Safety Center of Inquiry. Can we make postoperative patient handovers safer? A systematic review of the literature. Anesth. Analg 2012; 115 (1): 102–15. 22543067 10.1213/ANE.0b013e318253af4b PMC6152818

[5] Nagpal K , Arora S, Abboudi M, et al Postoperative handover: problems, pitfalls, and prevention of error. Ann Surg 2010; 252 (1): 171–6. 20505507 10.1097/SLA.0b013e3181dc3656

[6] Nagpal K , Arora S, Vats A, et al Failures in communication and information transfer across the surgical care pathway: interview study. BMJ Qual Saf 2012; 21 (10): 843–9. 10.1136/bmjqs-2012-000886 22773891

[7] Douglas RN , Stephens LS, Posner KL, et al Communication failures contributing to patient injury in anaesthesia malpractice claims. Br J Anaesth 2021; 127 (3): 470–8. 34238547 10.1016/j.bja.2021.05.030 PMC8563338

[8] Hughes HK , Serwint JR, O'Toole JK, Spector ND, Ngo TL. I-PASS adherence and implications for future handoff training. J Grad Med Educ 2019; 11 (3): 301–6. 31210861 10.4300/JGME-D-18-01086.1 PMC6570451

[9] Hughes TM , Dossett LA, Hawley ST, Telem DA. Recognizing heuristics and bias in clinical decision-making. Ann Surg 2020; 271 (5): 813–4. 31850992 10.1097/SLA.0000000000003699

[10] Wheeler DS , Sheets AM, Ryckman FC. Improving transitions of care between the operating room and intensive care unit. Transl Pediatr 2018; 7 (4): 299–307. 30460182 10.21037/tp.2018.09.09 PMC6212379

[11] Wright MC , Endsley MR. Building shared situation awareness in healthcare settings. In: Nemeth CP, ed. Improving Healthcare Team Communication. London: CRC Press; 2008.

[12] Bergman AA , Flanagan ME, Ebright PR, O’Brien CM, Frankel RM. “Mr Smith’s been our problem child today…”: anticipatory management communication (AMC) in VA end-of-shift medicine and nursing handoffs. BMJ Qual Saf 2016; 25 (2): 84–91. 10.1136/bmjqs-2014-003694 26221029

[13] Horwitz LI , Moin T, Krumholz HM, Wang L, Bradley EH. Consequences of inadequate sign-out for patient care. Arch Intern Med 2008; 168 (16): 1755–60. 18779462 10.1001/archinte.168.16.1755

[14] Wells CI , Varghese C, Boyle LJ, et al ‘Failure to rescue’ following colorectal cancer resection: variation and improvements in a national study of postoperative mortality: reducing mortality after colorectal surgery. Ann Surg 2022; doi:10.1097/SLA.0000000000005650. 35920564

[15] Starmer AJ , Schnock KO, Lyons A, et al Effects of the I-PASS Nursing Handoff Bundle on communication quality and workflow. BMJ Qual Saf 2017; 26 (12): 949–57. 10.1136/bmjqs-2016-006224 28679836

[16] Sheth S , McCarthy E, Kipps AK, et al Changes in efficiency and safety culture after integration of an I-PASS-supported handoff process. Pediatrics 2016; 137 (2): e20150166. 26743818 10.1542/peds.2015-0166

[17] Shahid S , Thomas S. Situation, background, assessment, recommendation (SBAR) communication tool for handoff in health care – a narrative review. Saf Health 2018; 4:7.

[18] Philibert I. Use of strategies from high-reliability organisations to the patient hand-off by resident physicians: practical implications. BMJ Qual. Saf 2009; 18 (4): 261–6. 10.1136/qshc.2008.031609 19651928

[19] Zjadewicz K , Deemer KS, Coulthard J, Doig CJ, Boiteau PJ. Identifying what is known about improving operating room to intensive care handovers: a scoping review. Am J Med Qual 2018; 33 (5): 540–8. 29374964 10.1177/1062860618754701

[20] McFarlane A. The impact of standardised perioperative handover protocols. J Perioper Pract 2018; doi:10/gh3427. 10.1177/1750458918775555 29726805

[21] Blazin LJ , Sitthi-Amorn J, Hoffman JM, Burlison JD. Improving patient handoffs and transitions through adaptation and implementation of I-PASS across multiple handoff settings. Pediatr Qual Saf 2020; 5 (4): e323. 32766496 10.1097/pq9.0000000000000323 PMC7382547

[22] Staggers N , Blaz JW. Research on nursing handoffs for medical and surgical settings: an integrative review. J Adv Nurs 2013; 69 (2): 247–62. 22764743 10.1111/j.1365-2648.2012.06087.x

[23] Street M , Phillips NM, Haesler E, Kent B. Refining nursing assessment and management with a new postanaesthetic care discharge tool to minimize surgical patient risk. J Adv Nurs 2018; 74 (11): 2566–76. 29943390 10.1111/jan.13779

[24] Street M , Phillips NM, Mohebbi M, Kent B. Effect of a newly designed observation, response and discharge chart in the Post Anaesthesia Care Unit on patient outcomes: a quasi-experimental study in Australia. BMJ Open 2017; 7 (12): e015149. 10.1136/bmjopen-2016-015149 PMC5778298 29203501

[25] Siddiqui N , Arzola C, Iqbal M, et al Deficits in information transfer between anaesthesiologist and postanaesthesia care unit staff: an analysis of patient handover. Eur J Anaesthesiol 2012; 29 (9): 438–45. 22569028 10.1097/EJA.0b013e3283543e43

[26] Milby A , Böhmer A, Gerbershagen MU, Joppich R, Wappler F. Quality of post-operative patient handover in the post-anaesthesia care unit: a prospective analysis. Acta Anaesthesiol Scand 2014; 58 (2): 192–7. 24355063 10.1111/aas.12249

[27] Halladay ML , Thompson JA, Vacchiano CA. Enhancing the quality of the anesthesia to postanesthesia care unit patient transfer through use of an electronic medical record-based handoff tool. J Perianesth Nurs 2019; 34 (3): 622–32. 30528308 10.1016/j.jopan.2018.09.002

[28] Pucher PH , Johnston MJ, Aggarwal R, Arora S, Darzi A. Effectiveness of interventions to improve patient handover in surgery: a systematic review. Surgery 2015; 158 (1): 85–95. 25999255 10.1016/j.surg.2015.02.017

[29] Wu DTY , Deoghare S, Shan Z, Meganathan K, Blondon K. The potential role of dashboard use and navigation in reducing medical errors of an electronic health record system: a mixed-method simulation handoff study. Health Syst (Basingstoke) 2019; 8 (3): 203–14. 31839932 10.1080/20476965.2019.1620637 PMC6896471

[30] Flemming D , Hübner U. How to improve change of shift handovers and collaborative grounding and what role does the electronic patient record system play? Results of a systematic literature review. Int J Med Inf 2013; 82 (7): 580–92. 10.1016/j.ijmedinf.2013.03.004 23628146

[31] Staggers N , Clark L, Blaz JW, Kapsandoy S. Why patient summaries in electronic health records do not provide the cognitive support necessary for nurses’ handoffs on medical and surgical units: insights from interviews and observations. Health Informatics J 2011; 17 (3): 209–23. 21937463 10.1177/1460458211405809

[32] Staggers N , Clark L, Blaz JW, Kapsandoy S. Nurses’ information management and use of electronic tools during acute care handoffs. West J Nurs Res 2012; 34 (2): 153–73. 21540355 10.1177/0193945911407089

[33] van Elten HJ , Sülz S, van Raaij EM, Wehrens R. Big data health care innovations: performance dashboarding as a process of collective sensemaking. J Med Internet Res 2022; 24 (2): e30201. 35191847 10.2196/30201 PMC8905474

[34] McGrow K. Artificial intelligence: essentials for nursing. Nursing 2019; 49 (9): 46–9. 10.1097/01.NURSE.0000577716.57052.8d PMC6716553 31365455

[35] Yang X , Chen A, PourNejatian N, et al A large language model for electronic health records. NPJ Digit Med 2022; 5 (1): 194. 36572766 10.1038/s41746-022-00742-2 PMC9792464

[36] Singhal K , Azizi S, Tu T et al Large language models encode clinical knowledge. 2022. Preprint at 10.48550/arXiv.2212.13138.

[37] Ke JXC , McIsaac DI, George RB, et al Postoperative mortality risk prediction that incorporates intraoperative vital signs: development and internal validation in a historical cohort. J Can Anesth 2022; 69 (9): 1086–98. 10.1007/s12630-022-02287-0 35996071

[38] Fritz BA , Cui Z, Zhang M, et al Deep-learning model for predicting 30-day postoperative mortality. Br J Anaesth 2019; 123 (5): 688–95. 31558311 10.1016/j.bja.2019.07.025 PMC6993109

[39] Mathis MR , Engoren MC, Williams AM, et al; BCIL Collaborators Group. Prediction of postoperative deterioration in cardiac surgery patients using electronic health record and physiologic waveform data. Anesthesiology 2022; 137 (5): 586–601. 35950802 10.1097/ALN.0000000000004345 PMC10227693

[40] Chiew CJ , Liu N, Wong TH, Sim YE, Abdullah HR. Utilizing machine learning methods for preoperative prediction of postsurgical mortality and intensive care unit admission. Ann Surg 2020; 272 (6): 1133–9. 30973386 10.1097/SLA.0000000000003297 PMC7668340

[41] Nakatani H , Nakao M, Uchiyama H, Toyoshiba H, Ochiai C. Predicting inpatient falls using natural language processing of nursing records obtained from Japanese Electronic Medical Records: case-control study. JMIR Med Inform 2020; 8 (4): e16970. 32319959 10.2196/16970 PMC7203618

[42] Komorowski M , Celi LA, Badawi O, Gordon AC, Faisal AA. The artificial intelligence clinician learns optimal treatment strategies for sepsis in intensive care. Nat Med 2018; 24 (11): 1716–20. 30349085 10.1038/s41591-018-0213-5

[43] Korach ZT , Cato KD, Collins SA, et al Unsupervised machine learning of topics documented by nurses about hospitalized patients prior to a rapid-response event. Appl Clin Inform 2019; 10 (5): 952–63. 31853936 10.1055/s-0039-3401814 PMC6920051

[44] Korach ZT , Yang J, Rossetti SC, et al Mining clinical phrases from nursing notes to discover risk factors of patient deterioration. Int J Med Inf 2020; 135: 104053. 10.1016/j.ijmedinf.2019.104053 PMC7103062 31884312

[45] Rossetti SC , Knaplund C, Albers D, et al Leveraging clinical expertise as a feature - not an outcome - of predictive models: evaluation of an early warning system use case. AMIA Annu Symp Proc 2019; 2019: 323–32. 32308825 PMC7153052

[46] Rossetti SC , Knaplund C, Albers D, et al Healthcare process modeling to phenotype clinician behaviors for exploiting the signal gain of clinical expertise (HPM-ExpertSignals): development and evaluation of a conceptual framework. J Am Med Inform Assoc 2021; 28 (6): 1242–51. 33624765 10.1093/jamia/ocab006 PMC8200261

[47] Cato KD , McGrow K, Rossetti SC. Transforming clinical data into wisdom: artificial intelligence implications for nurse leaders. Nurs Manag (Harrow) 2020; 51 (11): 24–30. 10.1097/01.NUMA.0000719396.83518.d6 PMC8018525 33086364

[48] Robert N. How artificial intelligence is changing nursing. Nurs Manag (Harrow) 2019; 50 (9): 30–9. 10.1097/01.NUMA.0000578988.56622.21 PMC7597764 31425440

[49] Seibert K , Domhoff D, Bruch D, et al Application scenarios for artificial intelligence in nursing care: rapid review. J Med Internet Res 2021; 23 (11): e26522. 34847057 10.2196/26522 PMC8669587

[50] von Gerich H , Moen H, Block LJ, et al Artificial intelligence-based technologies in nursing: a scoping literature review of the evidence. Int J Nurs Stud 2022; 127: 104153. 35092870 10.1016/j.ijnurstu.2021.104153

[51] Ting JJ , Garnett A. E-health decision support technologies in the prevention and management of pressure ulcers: a systematic review. Comput Inform Nurs 2021; 39 (12): 955–73. 34132227 10.1097/CIN.0000000000000780

[52] Hu Y-H , Lee Y-L, Kang M-F, Lee P-J. Constructing inpatient pressure injury prediction models using machine learning techniques. Comput Inform Nurs 2020; 38 (8): 415–23. 32205474 10.1097/CIN.0000000000000604

[53] Lindberg DS , Prosperi M, Bjarnadottir RI, et al Identification of important factors in an inpatient fall risk prediction model to improve the quality of care using EHR and electronic administrative data: a machine-learning approach. Int J Med Inf 2020; 143: 104272. 10.1016/j.ijmedinf.2020.104272 PMC8562928 32980667

[54] Dunn Lopez K , Gephart SM, Raszewski R, Sousa V, Shehorn LE, Abraham J. Integrative review of clinical decision support for registered nurses in acute care settings. J Am Med Inform Assoc 2017; 24 (2): 441–50. 27330074 10.1093/jamia/ocw084 PMC7651925

[55] Dowding D , Merrill JA, Barrón Y, Onorato N, Jonas K, Russell D. Usability evaluation of a dashboard for home care nurses. Comput Inform Nurs 2019; 37 (1): 11–9. 30394879 10.1097/CIN.0000000000000484 PMC6326881

[56] Forbes A , Surdeanu M, Jansen P, Carrington J. Transmitting narrative: an interactive shift-summarization tool for improving nurse communication. In: proceedings of 3rd IEEE Workshop on Interactive Visual Text Analytics. 2013. http://clulab.cs.arizona.edu/papers/textvis2013.pdf. Accessed July 7, 2021.

[57] Waller RG , Wright MC, Segall N, et al Novel displays of patient information in critical care settings: a systematic review. J Am Med Inform Assoc 2019; 26 (5): 479–89. 30865769 10.1093/jamia/ocy193 PMC6657276

[58] Rajkomar A , Oren E, Chen K, et al Scalable and accurate deep learning with electronic health records. NPJ Digit Med 2018; 1: 18. 31304302 10.1038/s41746-018-0029-1 PMC6550175

[59] Fejza A , Genevès P, Layaïda N, Bosson J-L. Scalable and interpretable predictive models for electronic health records. In: 2018 IEEE 5th International Conference on Data Science and Advanced Analytics, DSAA. 2018; 341–50; Turin, Italy.

[60] Lundberg SM , Nair B, Vavilala MS, et al Explainable machine-learning predictions for the prevention of hypoxaemia during surgery. Nat Biomed Eng 2018; 2 (10): 749–60. 31001455 10.1038/s41551-018-0304-0 PMC6467492

[61] Ronquillo CE , Peltonen L-M, Pruinelli L, et al Artificial intelligence in nursing: priorities and opportunities from an international invitational think-tank of the Nursing and Artificial Intelligence Leadership Collaborative. J Adv Nurs 2021; 77 (9): 3707–17. 34003504 10.1111/jan.14855 PMC7612744

[62] Zhou Y , Li Z, Li Y. Interdisciplinary collaboration between nursing and engineering in health care: a scoping review. Int. J. Nurs. Stud 2021; 117: 103900. 33677250 10.1016/j.ijnurstu.2021.103900

[63] Hunter J , Freer Y, Gatt A, Reiter E, Sripada S, Sykes C. Automatic generation of natural language nursing shift summaries in neonatal intensive care: BT-Nurse. Artif Intell Med 2012; 56 (3): 157–72. 23068882 10.1016/j.artmed.2012.09.002

[64] Chetta A , Carrington JM, Forbes AG. Augmenting EHR interfaces for enhanced nurse communication and decision making. In: proc. 2015 Workshop Visual Analytics in Healthcare. 2015; Association for Computing Machinery: 1–6; Chicago, IL.

[65] Abraham J , Bartek B, Meng A, et al Integrating machine learning predictions for perioperative risk management: towards an empirical design of a flexible-standardized risk assessment tool. J Biomed Inform 2023; 137: 104270. 36516944 10.1016/j.jbi.2022.104270

[66] Abraham J , King CR, Meng A. Ascertaining design requirements for postoperative care transition interventions. Appl Clin Inform 2021; 12 (1): 107–15. 33626584 10.1055/s-0040-1721780 PMC7904383

[67] Weinger MB , Slagle JM, Kuntz AH, et al A multimodal intervention improves postanesthesia care unit handovers. Anesth Analg 2015; 121 (4): 957–71. 25806398 10.1213/ANE.0000000000000670

[68] Braun V , Clarke V, Hayfield N, Terry G. Thematic analysis. In: Liamputtong P. ed. Handbook of Research Methods in Health Social Sciences. Singapore: Springer; 2019: 843–860. doi:10.1007/978-981-10-5251-4_103.

[69] Tong A , Sainsbury P, Craig J. Consolidated criteria for reporting qualitative research (COREQ): a 32-item checklist for interviews and focus groups. Int. J. Qual. Health Care 2007; 19 (6): 349–57. 17872937 10.1093/intqhc/mzm042

[70] Hilligoss B , Moffatt-Bruce SD. The limits of checklists: handoff and narrative thinking. BMJ Qual Saf 2014; 23 (7): 528–33. 10.1136/bmjqs-2013-002705 24694362

[71] Dowding DW , Russell D, Onorato N, Merrill JA. Technology solutions to support care continuity in home care: a focus group study. J Healthc Qual 2018; 40 (4): 236–46. 28885241 10.1097/JHQ.0000000000000104 PMC5832509

[72] Li RC , Smith M, Lu J, et al Using AI to empower collaborative team workflows: two implementations for advance care planning and care escalation. NEJM Catal 2022; 3 (4): CAT.21.0457.

[73] Schwartz JM , George M, Rossetti SC, et al Factors influencing clinician trust in predictive clinical decision support systems for in-hospital deterioration: qualitative descriptive study. JMIR Hum Factors 2022; 9 (2): e33960. 35550304 10.2196/33960 PMC9136656

[74] Barda AJ , Horvat CM, Hochheiser H. A qualitative research framework for the design of user-centered displays of explanations for machine learning model predictions in healthcare. BMC Med Inform Decis Mak 2020; 20 (1): 257. 33032582 10.1186/s12911-020-01276-x PMC7545557

[75] Helman S , Terry MA, Pellathy T, et al Engaging clinicians early during the development of a graphical user display of an intelligent alerting system at the bedside. Int J Med Inf 2022; 159: 104643. 10.1016/j.ijmedinf.2021.104643 PMC9040820 34973608

[76] Kannampallil T , Jones S, Abraham J. ‘This is our liver patient…’: use of narratives during resident and nurse handoff conversations. BMJ Qual Saf 2020; 29 (2): 135–41. 10.1136/bmjqs-2018-009268 31270253

[77] Diprose WK , Buist N, Hua N, Thurier Q, Shand G, Robinson R. Physician understanding, explainability, and trust in a hypothetical machine learning risk calculator. J Am Med Inform Assoc 2020; 27 (4): 592–600. 32106285 10.1093/jamia/ocz229 PMC7647292

[78] Ghassemi M , Oakden-Rayner L, Beam AL. The false hope of current approaches to explainable artificial intelligence in health care. Lancet Digit Health 2021; 3 (11): e745–50. 34711379 10.1016/S2589-7500(21)00208-9

[79] Ginestra JC , Giannini HM, Schweickert WD, et al Clinician perception of a machine learning-based early warning system designed to predict severe sepsis and septic shock. Crit. Care Med 2019; 47: 1477–84. 31135500 10.1097/CCM.0000000000003803 PMC6791738

[80] James MT , Har BJ, Tyrrell BD, et al Effect of clinical decision support with audit and feedback on prevention of acute kidney injury in patients undergoing coronary angiography: a randomized clinical trial. JAMA 2022; 328 (9): 839–49. 36066520 10.1001/jama.2022.13382 PMC9449791

[81] Bond RR , Novotny T, Andrsova I, et al Automation bias in medicine: the influence of automated diagnoses on interpreter accuracy and uncertainty when reading electrocardiograms. J Electrocardiol 2018; 51 (6): S6–S11. 30122457 10.1016/j.jelectrocard.2018.08.007

[82] Mosier KL , Skitka LJ, Heers S, Burdick M. Automation bias: decision making and performance in high-tech cockpits. Int J Aviat Psychol 1997; 8 (1): 47–63. 11540946 10.1207/s15327108ijap0801_3

[83] D’Hondt E , Ashby TJ, Chakroun I, Koninckx T, Wuyts R. Identifying and evaluating barriers for the implementation of machine learning in the intensive care unit. Commun Med 2022; 2 (1): 1–12. 36543940 10.1038/s43856-022-00225-1 PMC9768782

