# Supplementary material for: Potential uses of AI for perioperative nursing handoffs: a qualitative study
Source: JAMIA Open. 2023 Mar 16;6(1):ooad015. doi: 10.1093/jamiaopen/ooad015 (PMC10019806; doi:10.1093/jamiaopen/ooad015)
Supplement: ooad015_Supplementary_Data [file ooad015_supplementary_data.zip › merged_pacu_checklist.pdf]

Appendix S1: Handoff artifacts and protocols in use.

Figure S1.1: Preop nursing assessment sheet. This paper form is begun by an RN in the preop holding area while interviewing a patient, filling out the top four sections. The form is placed in the paper chart, where it accompanies the patient to the OR, PACU, and wards. The PACU RN fills out the “Intraop” and “Postop” sections. We found that this form was used in every OR-PACU handoff and almost every PACU-floor handoff.

CHART HAND OFF WORKSHEET

PT INFO

Name:

Case Number:

NKDA Allergy

PROCEDURE:

HISTORY

- ☐ HTN
- ☐ Asthma
- ☐ COPD
- ☐ Seizures
- ☐ Chronic pain
- ☐ GERD
- ☐ Cardiac history
- ☐ OSA - CPAP
- ☐ Parkinsons

Other:

DM Type 1
home insulin pump

DM Type 2

Fall Risk:
☐ yes ☐ no

Translator:

Isolation:

Implantable Device:

Outpatient: ☐ yes ☐ no

Family Member:

Belongings:

Phone number:

PREOP

VS:

Mental Status:

Skin Integrity:

Betablockers:
☐ yes ☐ no

Home Opioids:
☐ yes ☐ no

HCG Results:
☐ Positive ☐ Negative

Accucheck:

REGIONAL

PREOP

- ☐ Regional Block
- ☐ Spinal
- ☐ Epidural

POTENTIAL POST OP BLOCK

- ☐ Yes → Call Regional Anesthesia
- ☐ No → Reason: ____________________
- ☐ Maybe → Call Regional Anesthesia

Dermatome ____________________

Post-op Block ______________ Time: ________

RAS Nurse 314-362-3907

Regional Anesthesia phone: ____________________

INTRAOP

General	MAC	Spinal
ET	LMA	Epidural
Airway:	NP	ORAL

Insulin:

Complications

Skin

Versed	Zofran
Fentanyl	Pepcid
Dilaudid	Benadryl
Toradol	Decadron
Antibiotic	

Crystalloid

Colloid

EBL

U/O

Blood products

Relaxed / Reversed

Family Last Updated:

Positioning

POSTOP

IV Access

Fluids/Gtts

Skin Breakdown

Assessment:

Surgical Wound:

Concerns:

DRAINS
- JP
- CT
- HV
- Lumbar
- Ventric

COMPASS ORDERS
- CXR
- Hip/Knee/Pelvis
- PCA

LABS
- CBC
- BMP
- Coags
- Trops
- Glucose
- Other:

Misc/To Do:

Surgical MD Phone Number:

Sign Out Time:

Boarding Time:

Destination:

PLACE IN FRONT OF CHART

Figure S1.2: OR to PACU handoff protocol. A laminated printout of this document hangs by every PACU bay.

Handoff Critical Elements

Universal Handoff Process

- Receiving team begins critical monitor hookup
  - Report cannot start until hookup is complete
- Team members introduce themselves

Circulator

- Patient identification (confirm placement of wristband)
- Isolation type
- Position of patient intra-operatively
- Packing/retained items
- Family information/last update
- Belongings/valuables
- Special equipment
- Key intraoperative events
- "The thing I am most concerned about is..."
- "What other questions do you have for me?"

Surgical/Procedure

- Indication for surgery/procedure
- Baseline physical exam – neuro, demeanor, pertinent positives
- Expected post-op exam findings (known neurologic deficits, expected pulse check, etc)
- Drain/tubes/packing/ intentionally retained surgical items (location, number, types and labeled)
- Dressing/wound
- Complications
- Labs
- Imaging (including providers needing to review prior to discharge from PACU)
- Ability for alternate pain management – peripheral block, epidural, TAP
- Diet (including PO and per tube medications)
- Special considerations (positioning, hemodynamic/ flap check parameters if necessary)
- Contact information for service
- Patient disposition plan (home, floor, ICU)
- "The thing I am most concerned about is..."
- "What other questions do you have for me?"

Anesthesiology

- PMH/PSH
- Allergies
- Meds (specify which were taken prior to surgery relate to HTN, DM, Parkinson's, chronic pain)
- Baseline vitals; height, weight, pain
- Baseline labs of significanc[illegible]
- Airway
- Lines
- Procedures (blocks, spinals
- Fluids (EBL and blood or blood products)
- Paralytic status
- Labs
- Meds (vasopressors, last dose of antibiotics, other intraoperative medications and infusions)
- Key events (hemodynamic stability)
- Pain management for this patient will consist of...
- Code status (including any conversations about temporary suspensions of DNR or DNI)
- Orders to follow up on (labs blood glucose, CXR for ___
- "The thing I am most concerned about is..."
- "What other questions do you have for me?"

Figure S1.3: PACU to Ward handoff protocol. This document is the reverse side of Figure S1.2.

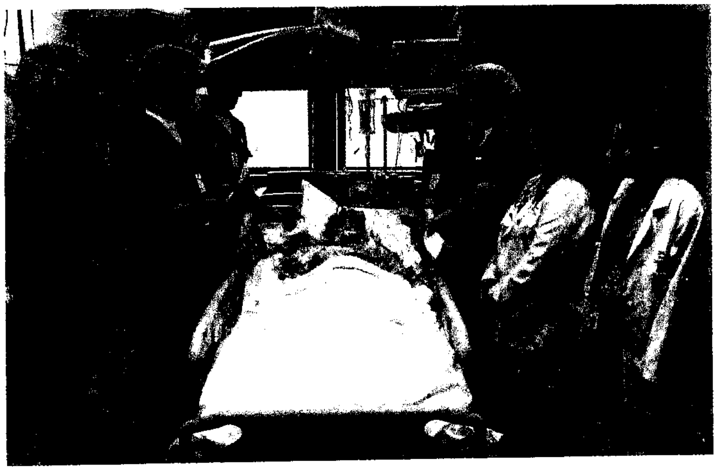

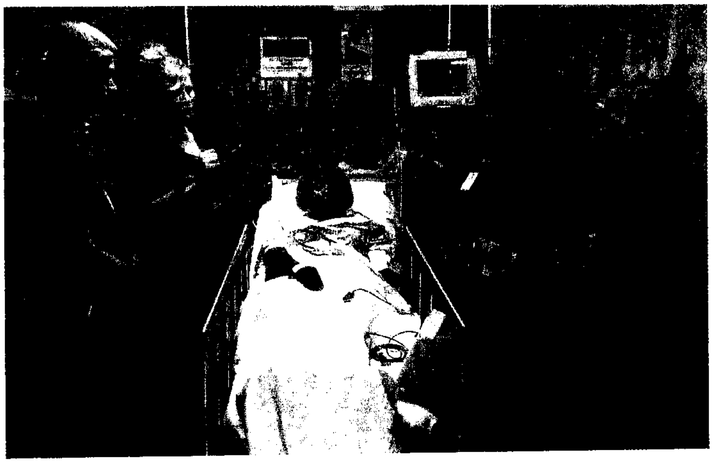

Patients Being Discharged From PACU

- Identification of patient and procedure
- Important past medical history
- Any complications intraoperative
- Any complications postoperative
- I and O's
- Labs/images results or pending
- Medications given and resolutions
- Regional blocks
- OSA risk
- Patient placement
- Telemetry ordered

BARNES JEWISH
Hospital
BJC HealthCare
