# Supplementary material for: Potential uses of AI for perioperative nursing handoffs: a qualitative study
Source: JAMIA Open. 2023 Mar 16;6(1):ooad015. doi: 10.1093/jamiaopen/ooad015 (PMC10019806; doi:10.1093/jamiaopen/ooad015)
Supplement: ooad015_Supplementary_Data [file ooad015_supplementary_data.zip › Shorter pacu interview.pdf]

PACU Nurse Interview Guide

Intro statement – review consent and study

Thank you so much for participating. To review the study consent document we went over, this will be an interview about handoffs in the PACU and how predictive analytics might fit into your workflow. This is an IRB approved study, and we will be recording the conversation and transcribing it. You can withdraw at any time, and I haven't offered your any compensation. Does that match your understanding?

Intro statement – topic

To set up context, do you primarily work in PACU or do you also work in preop or other areas? Do you work only at BJH or other hospitals as well?

1. What would you say the contribution of the PACU is to preventing postop complications?

I understand that when a patient comes from the OR, after they are hooked up to the monitor, and then the circulator, surgeon, and anesthesia provider give report.

2. How often is the handoff protocol fully followed? Are there any major omissions that happen?
3. Is there information that you routinely need to look for in Epic after handoff?
4. Is there information you wish was easier to get to?
5. Do you talk about patient specific risks of complications at handoff?

6. How does your monitoring and evaluation vary from patient to patient in PACU?
    a. Do you spend more time with patients or change what you're looking for based on their comorbidities or surgery?
    b. Does your communication with the surgery service or PACU anesthesia team vary based on comorbidities or surgery?

I'd like to ask a few questions about the communication to the floor. My understanding is that after the patient has met some PACU goals and anesthesia has signed them out, you call the floor nurse or transport with them and do a face to face handoff.

7. Is there a protocol for what needs to be communicated to the floor nurse?
8. Do you give any information to the floor provider (resident or NP)?
9. Are there questions the postop nurse frequently asks?
10. Do you talk about patient-specific risks of complications?
11. What do you think that we could do that would most improve the transfer of important information to the postop wards team?

I'd like to shift focus a bit and talk about the potential role of predictive analytics for PACU nurses. Epic and other investigators are looking into displaying machine learning predicted risks of common adverse events like pneumonia, delirium, and AKI into everyone's workflow. For context, you can assume that we are really only interested in more complicated cases with higher risk patients or surgeries.

12. Are there any specific adverse events where these calculated risks that would be useful for you to know?

a. if it was present at entry to the PACU, would it affect what you looked for in handoff?
b. would it affect communication with anesth / surgery while in PACU
c. do you think knowing elevated risks like these might affect what you're looking for with the patient?
d. If this sort of information was integrated to Epic or included as a print-out with the preop nursing sheet, would you refer to it in your handoff to the floor?

13. Would it be useful to you to have more general risks like length of stay, ICU admission, and death? How so?
14. Let's say that something like ICU admission risk was going to be added. Can you think where in your workflow it might be reasonable to look at?
15. Again assuming that these were going to show up, are there thresholds in any of these risks that make them relevant? Would you like to see the numbers, a graph, or a simplified presentation (like low, medium, high)?
    a. Would it be helpful to include some comparisons like an “average patient”, patients getting this surgery?
    b. Would it be helpful to know how the risks changed during the OR useful?
16. A feature that's also been suggested is the link between risk factors and predictive analytic risks. For example, that the risk of ICU admission was high because of anemia and low albumin. This can also be shown quantitatively (e.g. the risk of ICU admission increased by 3% due to the low hemoglobin). Do you think those would be useful?
    a. What about (LOS is high) because (pneumonia risk is high)

Ward Nurse Interview Guide

Intro statement – review consent and study

Thank you so much for participating. To review the study consent document we went over, this will be an interview about handoffs from the PACU and how predictive analytics might fit into your workflow. This is an IRB approved study, and we will be recording the conversation and transcribing it. You can withdraw at any time, and I haven't offered your any compensation. Does that match your understanding?

1. What units / areas do you normally work in?
2. I have a big picture question to get started thinking about the topic: what would you say the contribution of the ward nurse is to preventing postop complications?
3. Tell me briefly about what happens when a patient comes from the PACU.
   (if not specifically addressed)
   a. Is there a specific handoff protocol that is followed? What are the elements of it? What fraction of the time is it completely followed?
   b. What specific tasks do you need to accomplish right away when a patient arrives?
4. Tell me about the information you get from handoff with the PACU.
   a. Do you normally get any information from the surgery resident / np?
   b. Do you think this is fairly complete in terms of understanding what happened in the OR and PACU? Is there anything important that is sometimes missed?
5. Is there information that you routinely look for in Epic after handoff?
   a. Do you look at the surgery NP "handoff" document?
   b. Is there information you wish was easier to get to?
   c. Is there any information that you rely on the paper chart for?
6. For immediate postop patients, how does the monitoring vary from patient to patient?
   a. Do you spend more time with patients or change what you're looking for based on their comorbidities or surgery?
7. As far as variation in nursing interventions go, are there some that you have the leeway to decide them on your own, and some that you reach out to the NP / physician for changes to orders? Can you briefly given an example?
8. What communication does the surgical team routinely expect from you?
   a. Other than an emergency or forgotten orders, what would you call the surgery team for?
   b. What do they get in touch with you for?
   c. Do you talk about patient-specific risks?
9. *Skip if > 15 minutes.* If you handoff the patient to another nurse before shift change from time to time is there a specific protocol for nurse-nurse handoffs?
   a. How do you share information in this case? Is there Epic documentation that you look for when taking over from another nurse?

Another topic we'd like to discuss is predictive analytics. Epic and other investigators are looking into displaying machine learning predicted risks of common adverse events like pneumonia, delirium, and AKI into everyone's workflow. For context, you can assume that we are really only interested in more complicated cases with higher risk patients or surgeries.

These would be generated based on information from the preoperative assessment, medical history, medication list, intraoperative data, and PACU events. We want to hear what might be useful to you and ask a few questions about how it might be presented.

10. Are there any specific adverse events where these calculated risks would be useful for you to know? Some common suggestions are ICU admission, death, AKI, respiratory failure, delirium, length of hospital stay, readmission.
    a. Do you think very specific risks (like pneumonia) are more useful, or more general risk like length of stay?
11. Let's say that something like ICU admission risk was going to be added. Can you think where in your workflow it might be reasonable to look at?
12. If some of these risks were going to be shown, would it be useful to have a comparison like an "average patient" or just show the absolute risk?
13. Would you like to see the numbers, a graph, or a simplified presentation (like low, medium, high)? Would the change over the course of the hospitalization or surgery be useful to show?
14. A feature that's also been suggested is the link between risk factors and predicted risks. For example, that the risk of ICU admission was high because the albumin was low. This can be shown qualitatively (like, just a list of risk factors) or quantitatively (e.g. the risk of ICU admission increased by 3% due to the low hemoglobin). Do you think those would be useful?
15. If this sort of information was integrated to Epic would you refer to it in your handoff from PACU? Do you think that you would refer to it in your documentation? What about the list of identified risk factors?

Finally, we'd like to hear any thoughts you have on these issues that we didn't touch on.

16. What do you think that we could do that would most improve the transfer of important information to the postop nursing team?
