# Supplementary material for: Potential uses of AI for perioperative nursing handoffs: a qualitative study
Source: JAMIA Open. 2023 Mar 16;6(1):ooad015. doi: 10.1093/jamiaopen/ooad015 (PMC10019806; doi:10.1093/jamiaopen/ooad015)
Supplement: ooad015_Supplementary_Data [file ooad015_supplementary_data.zip › sup_table.docx]

**Supplement 5**

**Table S5**: Interview themes, subthemes, and supporting quotations.

|  | **Subtheme format: theme statement [linked subtheme]. Quote (stop words and verbal pauses removed).** | **Part ID** | **Ward**  **or**  **PACU** | **N** |
| --- | --- | --- | --- | --- |
| 1 | **Nurse understanding of patient condition guides care decisions:** |  |  |  |
| 1.a | *RN monitoring leads to early detection, mitigation of complications.* “Especially on the floor that I work on, it's primary nursing, so we do everything, assessments, post-op, vitals. We're the hands and eyes on the patient. If anything goes downhill, then we're the first ones to know to let the providers know and to take action.” | 9 | Both | 7 |
| 1.b | *RN understanding leads to changes in ongoing patient assessment.* “[I]t just depends on what the patient had done and what complications they were having, either pre-operatively or postoperatively. One of our major patients up on [ward] is the kidney transplants. So obviously, for them, my biggest concern is what's their urine output and what's their vitals. … Somebody who's had a major abdominal surgery, and they lost a lot of blood, what's their vitals and their labs looking like? … So it is just patient specific, the main worry or concern with each patient.” | 10 | Both | 11 |
| 1.c | *RN understanding of priorities changes important team communication.* “I know whenever the floor doctors come up and check on the patient, I tell them all this information and they're like, "Oh, I wasn't aware of this." And I'm like, "Okay. Well, here, I'm letting you know this now." … I feel like that is where there's a lot of miscommunication, and it takes a lot longer to be able to treat the patient” | 11 | Both | 8 |
| 2 | **Handoffs are important to nurse situational awareness, but multiple barriers reduce their effectiveness** |  |  |  |
| 2.a | *HO detail is important to understand patient condition* [see 2.d, 3.b, 3.c]. “[Handoff is important for] What to expect ... if it's a spine case, what kind of deficit the patient had pre op, [or] what they're going to expect after having, say, a craniotomy. ... But the big thing is just the basic history.” | 1 | Both | 5 |
| 2.b | *Problem-focused handoff frequently absent* [see 3.a]. “Occasionally the circulating nurse will say something. Very infrequently the surgical person does. Sometimes they'll say they're concerned about pain or concerned about bleeding. If there's some sort of surgical complication they'll go into more specifics about that. Anesthesia will usually talk about if there was a problem with anything anesthesia related if they want you to keep an eye out for anything.” | 8 | Both | 8 |
| 2.c | *Lack of shared priorities inhibit effective PACU-Ward nurse handoff* [see 3.a]. “Sometimes I don't care to hear some things and sometimes I do care. It depends on the patient's condition. ... There is some stuff which we maybe should care about but don't, like, were they an easy airway? It's one of the things they always say. … or gosh, 'They got two liters of crystalloid, one of colloid,' that just blows over my head.” | 10 | Ward | 9 |
| 2.d | *Incomplete preop leads to less effective handoffs* [see 3.a, 3.d]. “If the patient isn't in pre op for very long, that paper document isn't really filled out to completion. … The only thing I really look at it for is when I'm giving report to the floor. I will use what anesthesia has told me.” | 4 | PACU | 4 |
| 3 | **AI may address barriers to handoff effectiveness** |  |  |  |
| 3.a | *AI adds problems to assessment, discuss in handoff.* [see 2.b, 2.c] “If you see that these people are at a risk for an AKI or something, I would be more apt to ask about the urine output or their last [hemato]crit values. Or if it says they're at a higher risk for being admitted to the ICU, I'll be like, "Well, are they doing okay? Do they seem okay to you?" I'll be asking some questions if I see that they're at risk for these things.” | 5 | Both | 8 |
| 3.b | *AI leads to better understanding of disease severity.* [see 2.d] (regarding interpreting alert thresholds) “I think a red would be recurrent exacerbations, where somebody's ended up in the ER, ICU, or even just hospitalized because of a breathing issue. ... or syncopal episodes that have had a fall or heart rate drop.” | 1 | Both | 3 |
| 3.c | *AI triggers reassessment of important problems.* [see 2.c] (regarding a high calculated risk) “I think it makes you more present to the physical patient as well. Looking at them and looking at all their vital signs, their trends. And I think it might just make you a little bit more mindful rather than being focused so much on charting.” | 3 | Both | 5 |
| 3.d | *AI could help find relevant data.* [see 2.d] “If your scores were all low for all your risk assessments, I probably wouldn't even try and dive deeper into that. But if they were high, somewhere you could find more information on why they were high like, "Why does this patient score so high?" either in a flowsheet … or some sort of note.” | 10 | Both | 6 |
| 4 | **AI may augment nurse care decision making and team communication outside of handoff** |  |  |  |
| 4.a | *Team communication on risks is important for RN care planning, frequently absent* [see 1.c, 2.c, 4.b]. “There was one resident ... he would look for us and be like, 'Hey, these are the main things that we're looking for.' And I really liked that. I never thought about it, but yeah, that was one person that did it. No one else really does say what the specific complications they're looking for.” | 11 | Ward | 6 |
| 4.b | *AI identified problems / risks could lead to changes in team communication*. [see 4.a] “I'll be getting a report from PACU, and … I'll be like, 'I don't know. This patient doesn't seem like they're going to do very well on the floor. It seems they need to be monitored closely.' And sometimes you express your concerns to a doctor or a charge nurse, and they'll just be like, 'Oh, well, we'll see how they do when they get here.' … maybe if they had some sort of kind of solid, concrete evidence right there in the chart ... where some of these things that are a little bit harder to all put together in a firm way” | 5 | Both | 3 |
| 4.c | *Incomplete physician documentation inhibits nurse awareness* [see 3.d, 1.b, 3.c, 4.d]. “Yes, because a lot of the times, … their [physician document] was not complete. It just said … "deceased kidney donor. This is their blood type, and this is what happened." They're just getting it started.” | 11 | Ward | 8 |
| 4.d | *AI problems could lead to prioritization in monitoring and assessment.* [see 4.c, 4.a] (regarding communicating a high AI calculated risk) “I would hope so. … [be]cause the patient still should be a little more closely monitored even when they're up on the floor, so I feel like it would help the floor nurse to narrow down what she should really be monitoring for.” | 4 | Both | 7 |
| 4.e | *AI risks predictions could trigger specific nursing interventions*. "I would definitely say delirium would be a good one. We do see a lot of that. And we also see a lot of alcohol and drug withdrawal but was either not talked about or just unexpected. Especially for those patients, if we could monitor them with our CIWA protocol or … already have the Ativan and all the medications ordered, then we could prevent an acute event from happening or an emergency.” | 9 | Both | 4 |
| 5 | **EHR user experience and information overload are likely barriers to using AI** |  |  |  |
| 5.a | *Difficult to agree on AI placement in EHR*. No exemplar quote. |  | Both | 7 |
| 5.b | *Difficult to agree on AI presentation mode.* No exemplar quote. |  | Both | 9 |
| 5.c | *AI flags easy to ignore.* “If it was a popup message, it probably wouldn't be as nice to have than if it's on the side … because sometimes stuff just pops up all the time on the computer, and … as bad as it sounds, you just exit out of it because, usually, it's something that you just haven't charted.” | 5 | Both | 3 |
| 5.d | *Easy for AI to info overload.* (regarding AI presentation) “Less is more. Not too much of a thing. I feel like the heavier it is, the more people are going to be like, 'Oh, I don't have time for that.'” | 8 | Both | 4 |
