# Supplementary material for: Potential uses of AI for perioperative nursing handoffs: a qualitative study
Source: JAMIA Open. 2023 Mar 16;6(1):ooad015. doi: 10.1093/jamiaopen/ooad015 (PMC10019806; doi:10.1093/jamiaopen/ooad015)
Supplement: ooad015_Supplementary_Data [file ooad015_supplementary_data.zip › Appendix S3.pdf]

Appendix S3: Core study topics used as closed codes

Description of handoff process

- Process failures, common information difficulties
- Protocol use / awareness
- Anticipatory guidance topics
- Information sought / sought by others

Nurse decision making

- Variations in care
- Communication with team and autonomous decisions

Use of AI

- Willingness to use / characteristics of effective
- Use cases: specific recommendations, physician or handoff communication, other
- Specific risks worth predicting

Presentation of AI

- Preferred presentation
- Workflow placement
- Feature attribution / data seeking
